# Modeling the Encoding of Saccade Kinematic Metrics in the Purkinje Cell Layer of the Cerebellar Vermis

**DOI:** 10.3389/fncom.2018.00108

**Published:** 2019-01-10

**Authors:** Hari Teja Kalidindi, Thomas George Thuruthel, Cecilia Laschi, Egidio Falotico

**Affiliations:** The BioRobotics Institute, Scuola Superiore Sant'Anna, Pisa, Italy

**Keywords:** saccade adaptation, saccades, oculomotor control, cerebellum, oculomotor vermis, forward model

## Abstract

Recent electrophysiological observations related to saccadic eye movements in rhesus monkeys, suggest a prediction of the sensory consequences of movement in the Purkinje cell layer of the cerebellar oculomotor vermis (OMV). A definite encoding of real-time motion of the eye has been observed in simple-spike responses of the combined burst-pause Purkinje cell populations, organized based upon their complex-spike directional tuning. However, the underlying control mechanisms that could lead to such action encoding are still unclear. We propose a saccade control model, with emphasis on the structure of the OMV and its interaction with the extra-cerebellar components. In the simulated bilateral organization of the OMV, each caudal fastigial nucleus is arranged to receive incoming projections from combined burst-pause Purkinje cell populations. The OMV, through the caudal fastigial nuclei, interacts with the brainstem to provide adaptive saccade gain corrections that minimize the visual error in reaching a given target location. The simulation results corroborate the experimental Purkinje cell population activity patterns and their relation with saccade kinematic metrics. The Purkinje layer activity that emerges from the proposed organization, precisely predicted the speed of the eye at different target eccentricities. Simulated granular layer activity suggests no separate dynamics with respect to shaping the bilateral Purkine layer activity. We further examine the validity of the simulated OMV in maintaining the accuracy of saccadic eye movements in the presence of signal dependent variabilities, that can occur in extra-cerebellar pathways.

## 1. Introduction

Saccades are rapid eye movements, observed in primates, carried out to bring a selected spatial target into the center of the fovea. The saccadic eye movements are executed in the absence of visual feedback information, suggesting a contribution of internal estimates (that predict sensorimotor consequences) in generating accurate eye movements (Shadmehr et al., 2010). In addition, various factors such as the mechanical state of the oculomotor plant, behavior of the target stimuli (Aslin and Salapatek, 1975), and variable motivational states associated with the same target location (Jürgens et al., 1981; Xu-Wilson et al., 2009) result in variabilities in motor commands. Adaptation is necessary in the neural pathways forming the internal estimates to deal with the presence of definite functions of variability, and this has been a topic of high interest in the experimental and modeling aspects of sensorimotor control (Hopp and Fuchs, 2004).

Lesion and inactivation studies (Zee et al., 1976; Xu-Wilson et al., 2009) carried out in the posterior lobes VIc and VII of the cerebellar vermis, also known as oculomotor vermis (OMV), indicate a loss of accuracy and adaptation in the saccadic eye movements. This OMV regulated adaptation (Robinson et al., 2006), involves both online correction of sensory-motor signals in the saccade control system to deal with inter-trial variability (Takagi et al., 1998), and also long-term corrections for the changes in the mechanical properties of oculomotor plant (Ritchie, 1976; Optican and Robinson, 1980; Robinson, 1995). These observations imply that the OMV can take into account the current state of the oculomotor system, and produce necessary corrections for accurate saccadic eye movements. To achieve accuracy, the correction signals from the OMV output should bear a definite relationship with saccadic activity. Particularly, OMV output is transferred to the extra-cerebellar components in the saccade production, by means of projections of the vermal Purkinje cells (PCs) (that form the sole output of the cerebellar vermis) onto caudal fastigial nuclei (cFN). However, the organization of the PCs and cFN in the cerebellar vermis and their interactions with the extra-cerebellar regions, responsible for such error correction mechanisms have not been fully addressed.

Definite saccade related contextual information such as desired saccade displacement and progress of the eye toward target, has been observed to be encoded in the inputs carried to the OMV as mossy fiber (MF) information (Kase et al., 1980; Yamada and Noda, 1987; Ohtsuka and Noda, 1992). This mossy fiber information undergoes further processing in the granular layer of the OMV, before transmission to the PCs, whose activity is further regulated by the error information received from the climbing fibers (Ito, 1990). The activity of climbing fiber afferents has been observed to have definite modulation with respect to the visual error from inaccurate saccadic movements (Soetedjo et al., 2008). A large number of these Purkinje cells converge in an inhibitory manner, onto the cFN. The activity of these cFN could be further shaped by excitatory projections from mossy fibers, as indicated in Ohtsuka and Noda (1991). These cFNs are directly involved in providing correction signals derived from the OMV, to the other regions of saccade generation circuitry. The lateralized cFN in the direction of saccades produces an early burst of saccade, and the cFN in the opposite direction produce a late burst for a certain duration that is related to the end of saccadic movement (Ohtsuka and Noda, 1991; Fuchs et al., 1993). The objective of the saccade models (Dean, 1995; Schweighofer et al., 1996), following the early experimental observations has been to explain various neural mechanisms that could cause the observed cFN discharges, that ultimately produce saccadic trajectories. These models do not reproduce the PC patterns responsible for such control. Another model in Quaia et al. (1999) and Optican (2005), reproduces the driving and braking signals generated by the cFN activity, but does not provide a plausible representation of the PC discharges.

In the earlier experimental and modeling cases, the functional organization of the PCs of the OMV, responsible for such stereotypical cFN activity was not examined well enough. Even though earlier experimental recordings illustrated saccade related discharges in individual PCs of the OMV, no definite encoding of saccade metrics such as amplitude, speed, or motor related information was observed in the firing patterns of those Purkinje cell outputs. Moreover, the duration of the recorded PC activity does not subside with the end of saccadic movement. This leads to an interesting question of the manner in which PCs in the OMV could encode the eye motion. A recent model in Gad and Anastasio (2010) aims to explain shaping of the cFN activity as a result of separate populations of burst and pause PC activities. In this model, the oculomotor drive signal by contralateral cFN burst in the beginning of saccade, is generated by the combined excitation from the MF inputs with the sequential pause and burst activity of the inhibiting PC projections. However, this model did not examine the possibility of amplitude modulation in the cumulative PC population activity, with respect to various saccade target eccentricities.

Contrary to the previous experimental observations on individual bursting or pausing PC population activities, recent recordings (Herzfeld et al., 2015) suggest that combined activities of burst-pause PC populations converging onto a common cFN, predict real-time motion of saccadic eye movements. The key difference from the earlier observations was to cluster the PC based upon their respective climbing fiber projections from the inferior olive. Furthermore, each of these PC population cluster has been considered to project onto a single cFN. The net inhibitory input from these Purkinje cell populations onto the cFN is observed to encode the direction and speed of saccades via a gain field. These experimental recordings on the PC population activity, can present a transition in the understanding of the role of OMV in saccade adaptation. In this context, we propose to model the predictive encoding of saccade kinematic metrics observed in the Purkinje cell population responses, and its role in the endpoint control of saccadic eye movements. Before, it is important to elaborate the overall neuroanatomical connections involved in the saccade sensorimotor control.

### 1.1. Neuroanatomy

**Figure 1:**
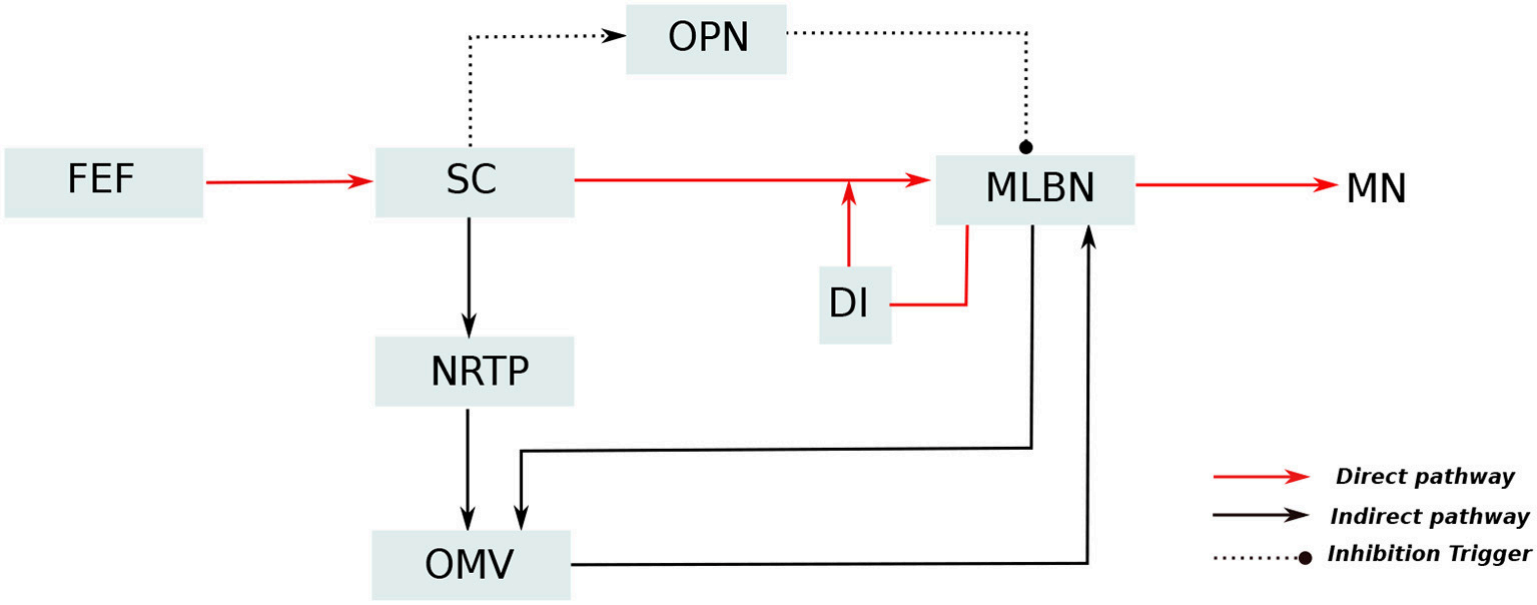
Simplified representation of the neuro-anatomical pathways involved in saccade production. The target information from the frontal eye fields (FEF) diverges into two distinct pathways for generation of motor control signal: The direct pathway, represented as red lines, passes through the superior colliculus (SC) which provides excitatory input drive to the medium lead burst neurons (MLBNs). A displacement integrator (DI) with gain lower than unity is employed to represent the inaccurate feedback information in case of vermal lesions. Additionally, the SC is hypothesized to provide static hold on the eye position by influencing the activity of omnipause neurons (OPNs), represented as dotted lines. The indirect pathway, represented as black lines, undergoes additional processing in the cerebellar oculomotor vermis (OMV), before projecting drive/brake signals to the MLBNs.

A simplified representation of the anatomical organization involved in saccade production, based upon major experimental studies (Robinson, 1972; Jürgens et al., 1981; Hepp and Henn, 1983; Scudder, 1988), is presented in Figure 1. In these anatomical connections, Medium-lead burst neurons (MLBNs) in the brainstem are the only directly responsible connections for delivering velocity commands to the putative premotor circuitry (Van Gisbergen et al., 1981). Information regarding the target eccentricity could be regarded to be transferred from the frontal eye fields to the superior colliculus (SC). The MLBNs receive inputs from the superior colliculus (SC) through three different pathways (Schweighofer et al., 1996; Quaia et al., 1999). In the *direct pathway*, the SC information is projected directly onto the MLBNs through unilateral excitatory connections. The *indirect pathway* involves a further modification of the SC activity in the lobuli VIc and VII of the OMV, transferred through the nucleus reticularis tegmenti pontis (NRTP). The third pathway involves inhibitory connections from the omnipause neurons (OPNs), that acts as static hold for the oculomotor plant until it receives a drive trigger from the SC region to initiate eye movement. The MLBNs are driven by dynamic motor error, which is the difference between a desired target displacement and current eye displacement. An estimate of this eye displacement could be considered to be derived from integrating the velocity commands generated in the MLBNs (Van Gisbergen et al., 1981). This velocity integration is represented by the displacement integrator (DI) component in Figure 1. During the inactivation of the OMV in the *indirect pathway*, the DI can be considered to provide an inaccurate estimate of eye position, hence resulting in saccade inaccuracies with a non-zero endpoint error (termed as saccade dysmetry). Several closely related interpretations have explained the nature of this inaccurate eye position estimate (Dean, 1995; Quaia et al., 1999; Optican, 2005). For our study of the OMV regulated endpoint control, the model proposed in Dean (1995), involving a local feedback loop with gain lower than one and agnostic to the origin of the eye position estimate, is suitable for simplification of control computations. As already described above, the focus of this current work lies in simulating the role of OMV in the *indirect pathway*, that is responsible for precision in steering the eyes toward a given target.

### 1.2. Contribution

We develop our current model with a major goal to simulate the encoding of saccade kinematic metrics, observed in the cumulative burst-pause responses in the Purkinje cell populations of the cerebellar vermis, that project onto common fastigial nucleus (Herzfeld et al., 2015). Our focus is to shed light on the underlying vermal and nucleus activities, by implementing simplest assumptions that could result in such action encoding. The first simplification we implement, is to consider a rate-based control approach for simulating the involved neural components, as explained in detail in the Methods section. This allows us to emulate the total input to each cFN (i.e., the net PC population projection onto cFN), and the output activity of the cFN responsible for generating the necessary motor commands. Furthermore, the PC layers are not comprised of purely burst or purely pause type activities, but contain combined burst-pause responses. This is inline with the hypothesis that the action encoding is observed in the total inhibitory activity onto the cFN from burst-pause Purkinje cell populations. The second simplification, as elaborated in the Methods section, is to consider the lateralized PC populations on each side to act exactly in opposite directions with respect to the motor command delivered to the oculomotor plant.

Our model demonstrates that, the cFN activity could be shaped by the cumulative responses from the burst-pause Purkinje cell populations. The PC information is further combined with excitatory projections from mossy fibers arising in the brainstem region, to form the net cFN activity. The cFN activity thus generated, provides necessary corrections to the saccadic movements. To simulate the ability of the cerebellar vermis to generalize the learning from different input contexts, we implement sparse connectivity in the granular layer units (simulated as rate based leaky-integrator units), with random inhibitory interconnections (Yamazaki and Tanaka, 2007a; Rössert et al., 2015; Bratby et al., 2016) and excitatory mossy fiber projections that carry the state of saccadic eye movement.

Sensory estimation, in the form of speed encoding, in the Purkinje layer facilitates the modulation of motor error input to the burst generating units in the brainstem region. This sensory estimation in the PC population response itself is shown in the model to result from the overall objective to reduce the visual target reaching error. The modulation of visual reaching error is facilitated by plastic connection strengths at the PF-PC synaptic connection site. Overall, our model demonstrates the role of OMV as a forward model estimator for sensory consequences of eye movement, backed by recent saccade experimental evidences.

With the same model, we illustrate the mechanism by which the presented OMV configuration can compensate for signal-dependent variabilities, associated with the extra-cerebellar pathways of saccade production. The results indicate that the OMV could provide appropriate compensations for variabilities, by means of its forward model mapping of the kinematic state of the eye movement to the necessary motor error corrections at the brainstem region.

## 2. Methods

**Figure 2:**
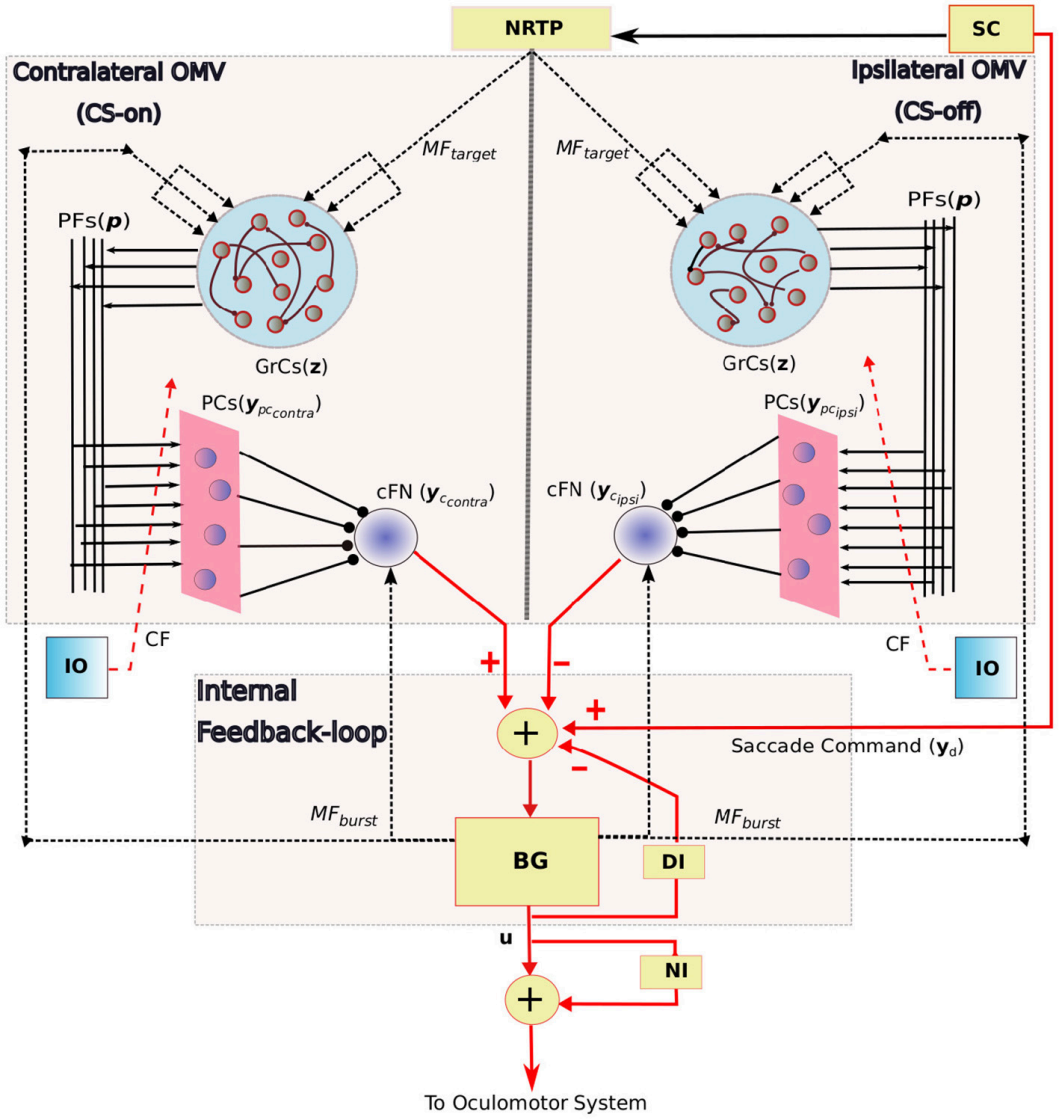
Schematic diagram showing the bilateral organization of the OMV and its connectivity with the internal feedback loop in saccadic production. The anatomical components in this saccade control system includes the *SC*, superior colliculus; *BG*, burst generating units to provide velocity commands *u* to the oculomotor system; *DI*, displacement integrator for providing inaccurate internal estimate of the eye position; *NI*, neural integrator that provides tonic discharge to hold the eye in place; Target information to the OMV is provided by *MF*_*target*_ signal, the state of the *BG* is provided by *MF*_*burst*_; *z* is the granule cell layer activity; *y*_*pc*_*contra*__ and *y*_*pc*_*ipsi*__ represent the bilateral PC population activity that receive same climbing fiber (*CF*) projections from the inferior olive (*IO*); *yc*_*contra*_ and *yc*_*ipsi*_ represent the net bilateral cFN activity. Arrowed projections indicate excitatory connections and circled projections are inhibitory. *MF* and *CF* inputs to the OMV are represented using dotted lines, connections to the grouped internal feedback loop are represented in red colored lines. PF-PC weights are the variable connection parameters.

The overall neuronal circuitry of our model is presented in Figure 2, and the main features include: 1. An internal feedback loop based on the grouped brainstem properties, that generates high magnitude bursts as velocity commands to the premoto-neuron circuitry. 2. Bilateral OMV region, that compensate for the errors and variabilities associated with horizontal eye movements.

The key components of saccade production system are already outlined in Figure 1. The oculomotor system is composed of complex and distributed neuronal circuitry with directional selectivities. Each of the component does not need an explicit neuronal representation for analyzing the OMV structure under study, and several previous works have made simplified implementations for the study of specific regions (Dean, 1995; Schweighofer et al., 1996; Optican, 2005). Hence, we make several simplifications in our saccade model. The key feature of the OMV adaptation is the active modulation of the motor error delivered to the brainstem MLBNs, by the OMV outputs through the cFN, by means of plasticity in the PF-PC synaptic weights. We approximate the distributed brainstem burst neurons to a grouped control block as detailed in the internal feedback loop subsection below. The SC is replaced by a dummy neuron unit similar to Dean (1995), that outputs a step signal with firing-rate proportional to the desired saccade amplitude (1 Hz = 1° desired saccade amplitude). This does not alter the analysis on the OMV because we focus on the OMV output responsible for eye movement correction, without including possible OMV to SC modifications. Moreover, the inputs to the OMV, as described in this section, are set to be physiologically plausible. The NRTP is just used as a relay. Furthermore, we employ a switch mechanism activated after a fixed time duration from the saccade beginning, to cut-off the activities of the brainstem and OMV inputs, and hold the eye at the reached position at the cut-off time. This switch relaxes the model from having to send separate eye-hold signals to the omnipause neurons (OPNs). Without loss of generality, we simulate horizontal saccades toward targets located in rightward direction.

### 2.1. Internal Feedback Loop

The shaping of the brainstem output is determined by the contributions from desired displacement command (**y**_*d*_), cerebellar output (**y**_*c*_), and the displacement integrator that performs imperfect integration of its own activity (**u**) over time, as presented in Equation (1). The distributed connections of excitatory and inhibitory burst neurons (EBN, IBN) in the brainstem are simplified in a grouped control block, for emulating the average burst behaviors observed during the inactivation of cerebellar compensation for the saccade control. The net output from the brain stem is devised to represent the burst properties of the involved neurons as presented in Equation (1), similar to the approach followed in Van Gisbergen et al. (1981) and Dean (1995) for brainstem representation,

(1) $$\begin{array}{l} \text{u}\left(t\right)=A\left(1-\exp^{-\left({\text{y}}_{c}+{\text{y}}_{d}-k\int \text{u}dt\right)/\sigma }\right) \end{array}$$

where, **u**(*t*) represents the magnitude of the burst at time *t*, *A* is the amplitude of the burst and fixed to a value of 1, 100*Hz*. The parameter *k* varies the end-point error in the absence of cerebellum. *k* = 0 results in continuous increase in the eye position, and *k* = 1 causes the eye to reach the target displacement **y**_*d*_ with significantly lower speed. The intermediate values *k* ∈ (0, 1) result in non-zero reaching error with no control in the eye speed. For the monkey oculomotor system, the value of *k* is considered to be a fixed parameter equal to 0.72 and σ is fixed at a value 16 (Dean, 1995).

### 2.2. Oculomotor Vermis

The indirect pathway of saccade motor control includes the SC-NRTP-OMV-cFN connections as shown in Figure 2, with OMV as the key neuronal processing site. Contrary to many of the previous modeling studies (Dean, 1995; Quaia et al., 1999; Gad and Anastasio, 2010), we include the granular layer computations as an essential component in shaping the PC population activity. It is important to note that the PC layer activity described in this model represents the total population response projecting onto the cFN. Hence, the simulated PC layer does not represent an individual biological Purkinje cell, but represent the total PC projections onto a given cFN.

#### 2.2.1. Input Information

In the context of eye movement controllability in the saccade control scheme, the OMV requires the target displacement information, and an estimate of the progress of eye movement toward the target location. The NRTP activity contains information about the desired target displacement, and acts as the source of target information to the OMV(Crandall and Keller, 1985; Ohtsuka and Noda, 1992). These MFs from the NRTP region, MF_*target*_, are set to transmit the target displacement information, **y**_*d*_, in the form of an amplitude-proportional activity. The experimental recordings from the MFs originating from the NRTP in Ohtsuka and Noda (1991, 1992) indeed show the existence of such MF types, with definite relationship between peak firing rates and the saccade amplitude. The target related MF activity is simulated to build-up gradually before the movement initiation, *t*_*ON*_, to mimic the long-lead characteristics expressed as $u_{target}\left(t|t\leq t_{ON}\right)=\text{λ}y_{d}e\frac{-{\left(t-t_{ON}\right)}^{2}}{\alpha^{2}}$ represents the final value reached by the build-up and α is the spread. Further the MF_*target*_ activity sustains at the value λ**y**_*d*_ from *t*_*ON*_ until the end of the eye movement.

On the other hand, the progress of eye movement information to the OMV does not need to come directly from the visual or proprioceptive sensors. Indeed, the experimental results on monkeys in Lewis et al. (2001), show an independence of on-line saccade eye control from the proprioceptive information (for linear range of eye movement). In our model, the OMV receives brainstem burst related efference copy information, **u**_*burst*_, through mossy fibers *MF*_*burst*_. In regard to this *MF*_*burst*_ activity, two separate sources of MF activity are found in the previous literature. Experimental recordings (Kase et al., 1980; Yamada and Noda, 1987), and modeling studies in Schweighofer et al. (1996) and Quaia et al. (1999) propose the brainstem burst information related MFs to be originating directly as bilateral projections from the brainstem PPRF region. On the other hand, Gad and Anastasio (2010) considered the MF to be derived from the burst-tonic signal provided from SC projections through NRTP structure. Nevertheless, the important aspect that is agnostic to specific projection sites, is the agreement regarding the presence of the brainstem burst information, **u**(*t*), in the MF projections onto the OMV. The *MF*_*burst*_ activity is simulated as feedback from the brainstem burst generator during the movement.

#### 2.2.2. Granule Cell Activity

The overall mossy fiber afferents carry the activity **m**(*t*) = (**u**_*target*_(*t*), **u**_*burst*_(*t*)) at a given time *t*. This information should be used by the OMV to compute the necessary compensations, **y**_*c*_ as depicted in **Figure 6**. These MF afferents excite the downstream granule cells whose activity is computed as follows:

(2) $$\begin{array}{l} \tau \dot{\text{z}\left(t\right)}={\left[-\text{z}\left(t\right)+\text{S}\left(t\right)\right]}^{+} \end{array}$$

where, τ is the time constant, **z**(*t*) is the vector of *N* granule cell activities at time *t*, **S**(*t*) is the net synaptic projection on to each granule cell at time *t* given by

(3) $$\begin{array}{l} \text{S}\left(t\right)=f\left({\text{w}}_{mf-GrC}\text{m}\left(t\right)-\rho \text{w}\text{z}\left(t\right)\right) \end{array}$$

where, **w**_*mf*−*GrC*_ represent the vector strengths of *N* mossy fiber projections onto the granular layer, **w** represents the *N* × *N* matrix of connection strengths in the granule layer. The weights **w** are strictly positive so that the term −ρ**wz**(*t*) is always negative, and hence results in inhibitory connection as represented by Equation (3). Separate populations of Golgi inter-neurons are not included in this model. Moreover, both **w**_*mf*−*GrC*_ and **w** do not contain any manually set values of connection weights, but are a set as sparse and random distribution for having a stable granule layer activity. *f* is considered to be a sigmoid function. An additional parameter ρ referred as the spectral radius, is used to limit the maximum weights in the granule layer to ensure the stability of the granular layer dynamics. As the exact connectivity patterns in the granule layer are not yet clear from experimental recordings, this sparse-random connectivity should serve as a reasonable and minimalistic assumption for gain and timing control studies (Yamazaki and Tanaka, 2007b). This kind of granule cell representation is known to carry out an appropriate signal expansion of the mossy fiber inputs in a number of computational models (Yamazaki and Tanaka, 2007a, 2009; Rössert et al., 2015).

#### 2.2.3. PC and cFN Activity

The granule activity (**z**) is transmitted by means of PF connections (**p**) to the PCs, with **w**_*pf*−*pc*_ connection strength vector of length *N*. The important concept underlying the objectives of this work is the sorting of burst-pause PC populations onto bilateral cFN, so that they can be enabled to be separately tuned for the directions of task space error. In experimental terminology, the PC units that show maximum firing for a given error direction can be labeled as CS-on in that particular direction, and the opposite direction (at 180°) as CS-off (Herzfeld et al., 2015). Furthermore, these CS sorted PC populations (combined burst-pause PCs) are found to be lateralized, with the CS-on sorted PC layer available on the contralateral side, and CS-off PC layer available on the ipsilateral sides of the eye movement. We have simulated burst-pause PC populations on contralateral and ipsilateral sides of the eye movements as shown in Figure 2.

We approximate the PC layer to contain a sum of all the projecting PFs weighted by the respective PF-PC connection strengths. The state of the PCs is combined with the mossy fiber strength onto the fastigial nucleus (cFN). **w**_*pc*−*cFN*_ represent the strength of PC-cFN connections, and **w**_*mf*−*cFN*_ represent the connection from MFs to cFN. Hence,

(4) $$\begin{array}{lll} {\text{y}}_{pc_{contra}}\left(t\right) & = & {\text{w}}_{pf-pc_{contra}}\text{z}\left(t\right)\\ {\text{y}}_{pc_{ipsi}}\left(t\right) & = & {\text{w}}_{pf-pc_{ipsi}}\text{z}\left(t\right) \end{array}$$

(5) $$\begin{array}{lll} {\text{y}}_{c_{contra}}\left(t\right) & = & -{\text{w}}_{pc-cFN_{contra}}{\text{y}}_{pc_{contra}}\left(t\right)+{\text{w}}_{mf-cFN_{contra}}\text{u}\left(t\right)\\ {\text{y}}_{c_{ipsi}}\left(t\right) & = & -{\text{w}}_{pc-cFN_{ipsi}}{\text{y}}_{pc_{ipsi}}\left(t\right)+{\text{w}}_{mf-cFN_{ipsi}}\text{u}\left(t\right) \end{array}$$

The negative sign on the PC contribution to cFN activity in the above equation represents the inhibitory projections of PCs onto the cFN. Bilateral cFN activities described in the above equation affect the total motor command delivered to the oculomotor system through their projections onto the MLBNs. The contralateral cFN adds to the saccade command during the beginning of the movement by its excitation of the ipsilateral MLBNs. The ipsilateral cFN provides a braking signal at the end by means of a late burst and subsequent excitation of the contralateral MLBNs (Fuchs et al., 1985). The motor command to the oculomotor plant, delivered through motor neurons, is the net difference between the contralateral and ipsilateral MLBNs, simulated as one grouped unit. As depicted in Figure 2, the equivalent control representation of this anatomical detail is that the contralateral cFN adds a positive command to the eye movement and the ipsilateral cFN provides a negative command. If we assume that the **w**_*mf*−*cFN*_ and **w**_*pc*−*cFN*_ are constant, the PC populations are responsible for the driving and braking commands in the opposite manner as that of their respective cFN. Overall, the net contribution by the bilateral cFNs is considered as follows,

(6) $$\begin{array}{l} {\text{y}}_{c}\left(t\right)=r_{1}{\text{y}}_{c_{contra}}\left(t\right)+r_{2}{\text{y}}_{c_{ipsi}}\left(t\right) \end{array}$$

where *r*_1_ and *r*_2_ signify the responsibility of each cFN response to the motor command. Specifically in this paper the values used are *r*_1_ = 0.02 and *r*_2_ = −0.02. Furthermore, **w**_*pf*−*pc*_ are considered to be the only sites of plasticity in this model.

### 2.3. Learning Criterion

Contrary to the approach of adjustment of the free parameters to fit the experimental data for specific stimuli conditions, our objective is to model the experimental observations as emergent phenomena of task error reduction.

Traditionally, saccades have been considered to minimize the endpoint variability. The information from the climbing fiber neural correlates (Soetedjo et al., 2008, 2009) suggest that, the estimate of the discrepancy between the desired and the observed sensory states is the likely candidate to regulate saccade eye movements. Such cost considerations can be observed in several modeling studies (Chen-Harris et al., 2008; Saeb et al., 2011), without emphasis on the OMV activity. In this work, we employ the following cost computation for generating saccadic movements.

(7) $$\begin{array}{l} \begin{array}{l} J=\sum_{t=0}^{T}\left|\Delta g\left(t\right)\right|+\gamma w_{pf-pc}^{2}\\ \text{such that},\;\Delta g\left(t\right)=\Delta y_{d}\left(t\right)-\Delta y_{e}\left(t\right) \end{array} \end{array}$$

where *J* is the cumulative cost accumulated by the end of the saccadic movement, Δ**g**(*t*) is the gaze error between desired eye displacement (**y**_*d*_) and the original eye displacement (**y**_*e*_) at time *t*, **w**_*pf*−*pc*_ represent the adaptive synaptic connection strengths of the bilateral OMV, and γ represents the relative significance of weight regularization compared to the gaze accuracy represented by the first term.

The PC activity for a given MF input can be modulated by changing the PF-PC connection strengths. This results in downstream cFN activity modulation. In light of different contributions of the bilateral cFNs to the ongoing motor command as given in Equation (6), it is worthy to note that considering different proportions of *r*_1_ and *r*_2_ can result in different types of cFN responses—for example: only burst, only pause, burst before pause, or pause before burst responses. The exact kind of responses elicited are dependent upon the physiological constraints on the PCs and cFNs, such as the relative distributions of each type of cells, and their discharge properties. These additional physiological details can be added as constraints on the cost function depicted in Equation (7).

## 3. Results

We present the simulation results in four parts. In the first part, we present the saccadic trajectories and how they are related with physiological evidences on monkeys. In the second part, we illustrate the emergence of PC layer activity from our model, and its predictive encoding of saccade kinematic metrics such as amplitude and speed. As described in previous sections, the simulated PC layer activity represents the total amount of inhibitory activity projecting onto each cFN, by the combined burst-pause PC populations organized by their common complex-spike/error property. We present a comparison with the experimental evidences described in Herzfeld et al. (2015). This is followed by the portrayal of the vermal and cFN activities. In the final part, we illustrate the ability of the same model to compensate for variabilities associated with the saccadic eye control.

The simulations are carried out to have adaptive weight updates at the PF-PC connections **w**_*pf*−*pc*_*contra*__ and **w**_*pf*−*pc*_*ipsi*__. The eye plant with the orbital tissue is approximated by a second order plant of the form,

(8) $$\begin{array}{l} k_{1}\ddot{\theta }+k_{2}\dot{\theta }+k_{3}\theta =m\left(t\right) \end{array}$$

Where, θ(*t*) is the eye position and *m*(*t*) is the net command or motor neuron firing rate imparted to the eye plant at time *t*. *k*_1_, *k*_2_, and *k*_3_ are the plant constants and set to the values 0.003 and 0.6 and 4, respectively. All the parameters considered for simulations are presented in Table 1. The adaptation trials consist of delivering motor commands to the oculomotor plant (the same monkey eye model as considered in Dean, 1995), through the mentioned *direct* and *indirect pathways*, resulting in specific eye movement toward the given target locations. These target locations are randomly chosen horizontal eye displacements between 4 and 20°. The endpoint error information obtained at the end of each trial is used to update the modifiable weights in the control loop, using *fmincon* optimization toolbox in MATLAB/SIMULINK. This toolbox uses finite difference based search for estimating the value of gradients at each iteration of optimization, hence alleviating the requirement to supply analytic incremental gradient update rule (while ensuring the applicability of incremental weight updates). Each adaptation trial is run for *T* = 0.4*s*, and the weight update is carried out until there is saturation in the cumulative endpoint error. As the properties of the reservoir neurons affect the shape of the produced granular layer activities and hence the overall PC layer output, instead of considering hand-set values of granule cell time constant τ and spectral radius ρ, we input these as variable parameters in the optimization loop. The values of τ over simulation trails is obtained to be close to 20*ms*, and the value of ρ is obtained to be equal to 0.5. The regularization coefficient, γ, presented in Equation (7) is determined empirically and fixed at the value 0.0001, such that these values emulate the PC population activity shape derived from electrophysiological observations (Herzfeld et al., 2015). All the simulated population responses depicted in the results below were obtained with the calibration variables **w**_*pc*−*cFN*_*contra*__ = **w**_*pc*−*cFN*_*contra*__ ≈ 20, λ = 2, *r*_1_ = 0.02, and *r*_2_ = −0.02 as presented in the methods. Biological recordings for the typical range of various synaptic strengths in the OMV can facilitate appropriate tuning of these calibration variables.

**Table 1:** Model parameters.

**Parameter**	**Value**
*k*	0.72
*k*_1_	0.003
*k*_2_	0.6
*k*_3_	4
*A*	1, 100*Hz*
σ	16
λ	2
α	0.015*ms*
ρ	≈0.5
τ	20*ms*
*w*	∈[0, 0.2]
*w*_*mf*−*GrC*_	∈[−30, 30]
*w*_*pc*−*cFN*_*contra*__	≈20
*w*_*pc*−*cFN*_*ipsi*__	≈20
*r*_1_	0.02
*r*_2_	−0.02
*T*	150*ms*
γ	10^−4^

*Numerical quantities of all the fixed parameters presented in the methods*.

### 3.1. Saccade Adaptation Characteristics

As the brainstem and displacement integrator block was simulated to generate eye movements in the absence of cerebellum, the initial movement with no cerebellar contribution results in default overshooting of the eye from a given target. We use adaptation trials to adjust the **w**_*pf*−*pc*_ connection strengths, for the OMV module to be able to compensate for these imprecise eye movements. This involves providing a target saccade command to the control loop, and recording the visual error associated with the movement. Subsequently the PF-PC weights are adjusted based upon the sensory error according to the learning criterion described in the Methods section. This procedure is repeated until accurate saccades are executed.

**Figure 3:**
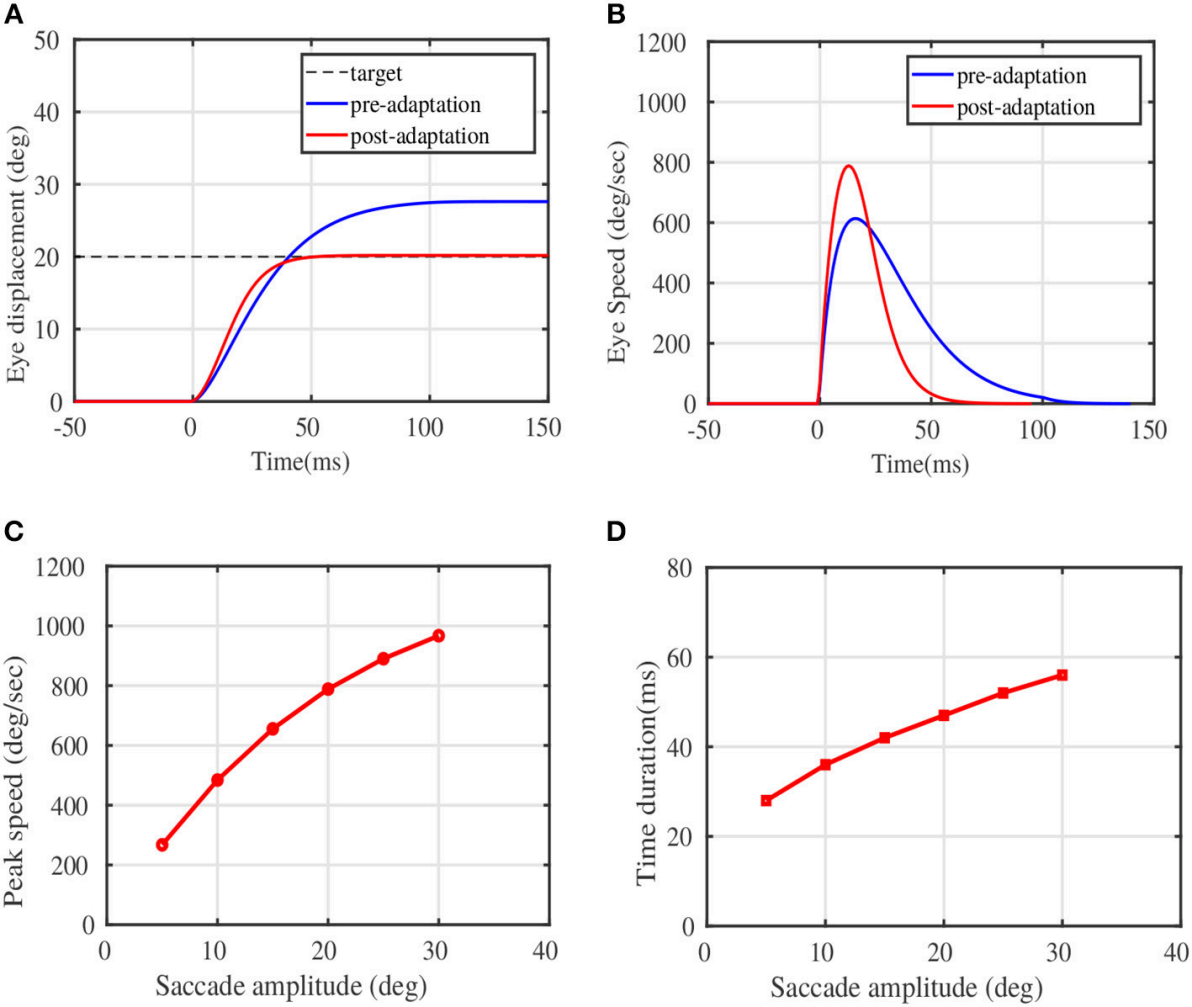
Adaptation results on sample test targets. **(A)** The displacement of eye position plotted against the movement time in milliseconds for a given test target of 20°. **(B)** The eye speed trajectory in deg/sec for the same test target of 20°. **(D)** Peak Eye speeds at various simulated target displacements post adaptation. **(C)** The duration of saccades against magnitude of the target displacements post adaptation. Comparison could be made from experimental observations on monkeys, shown in Figure 10 of Van Gisbergen et al. (1981), and in Figure 5 of Fuchs (1967).

Figure 3 illustrates the modulation of the eye movement trajectories before and after the adaptation trials. Figure 3A depicts the pre and post adaptation eye displacement characteristics for a given target displacement of 20°, while Figure 3B depicts the typical speed modulation. The x-axis in both plots refer to the saccade duration, with the eye movement initialized at *t* = 0*ms*. The peak speeds corresponding to target locations in the testing phase(post adaptation) are presented in Figure 3C. Figure 3D depicts the duration of the learnt eye movements for the presented target displacements. Both the speed-amplitude and duration-amplitude relationships, are in close alignment with the main sequence saccade patterns in monkeys (see Figure 5 of Fuchs, 1967 and Figure 10 of Van Gisbergen et al., 1981).

### 3.2. Encoding of Action in the PC Population Activity

**Figure 4:**
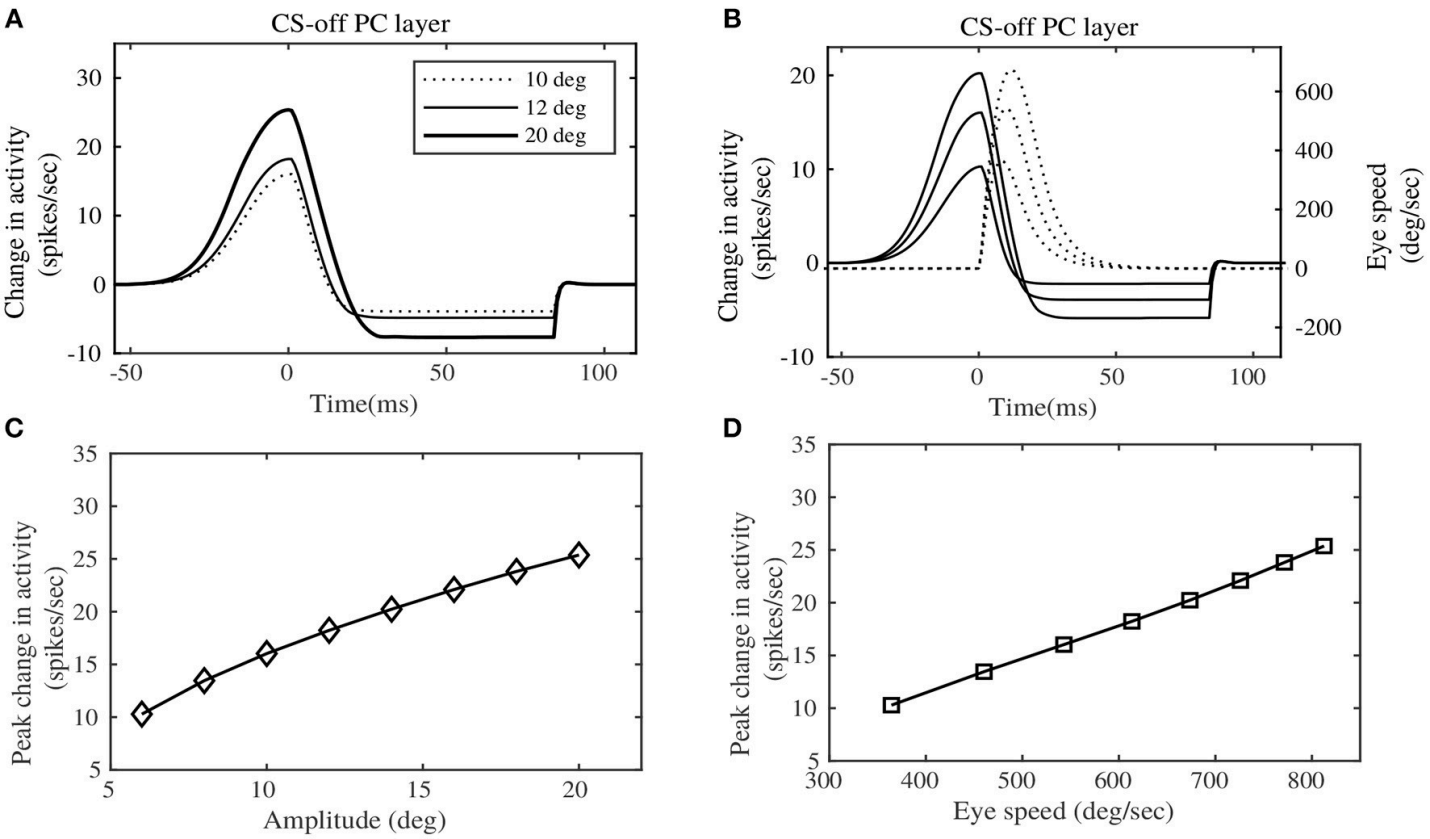
Eye motion information observed in the Purkinje layer activity. **(A)** Change in the CS-off PC layer activity at simulated target displacements (10, 12, and 20°). **(B)** Change in the CS-off PC layer activity at different speeds of Eye movement. **(C)** Peak change in the CS-off PC layer activity showed a slightly curved relationship with saccade amplitudes. **(D)** Peak change in the CS-off PC layer activity showed a linear relationship with saccade speed. Comparison could be made from experimental observations on rhesus monkeys, indicated by Figure 3 of Herzfeld et al. (2015).

Experimental results in Herzfeld et al. (2015) indicate a definite relationship of the PC population activity, sorted by their CS property, with saccade speeds and amplitudes. These CS sorted PC populations have been found to be anatomically lateralized. As already described in the Methods section, the contralateral PC layer can be considered to be the CS-on direction, ipsilateral PC layer could be considered to be the CS-off direction. Hence the terms CS-on and CS-off are used interchangeably to indicate the contralateral and ipsilateral PC layers respectively. We depict the net total population activity of the CS-off PC layer, in relation to saccade speed and amplitude in Figure 4 (that can be compared with the experimental observations in Figure 3 of Herzfeld et al., 2015). Figure 4A shows the change in the total activity of the CS-off PC layer, from baseline (considered at zero of the ordinate) for horizontal saccades of different amplitudes, made in the rightward direction. This CS-off PC layer activity is characterized by an early burst, with an onset prior to the initiation of saccadic movement (represented as dotted line in the figure at *t* = 0*s*), followed by relatively milder dip in the activity below baseline, that continues until the MF activity persists. The size of the PC layer activity is observed to increase with the size of the saccade amplitude (shown for 10, 12, and 20°). Although, the experimental observations in Herzfeld et al. (2015) illustrate a linear relationship between the CS-off PC population activity and saccade amplitude (*R*^2^ = 0.93, *P* < 10^−5^), in the simulations we observe a non-linear relationship (as indicated in Figure 4C). This can be because we perform simulations in a larger target range 4–20°, while the experiments were performed between 10 and 15°, where linearity could be observed.

Furthermore, the CS-off PC layer activity can be observed to be proportional to the saccade speeds (shown in Figure 4B). The CS-off PC layer activity reaches its peak before the peak eye speed, hence providing a predictive encoding of the saccade speed. The peak change in CS-off PC population activity is related linearly to the saccade speed associated with different target placements (as shown in Figure 4D), which corroborates the experimental observations (*R*^2^ = 0.98, *P* < 10^−7^).

### 3.3. Vermal and Fastigial Nucleus Activity

**Figure 5:**
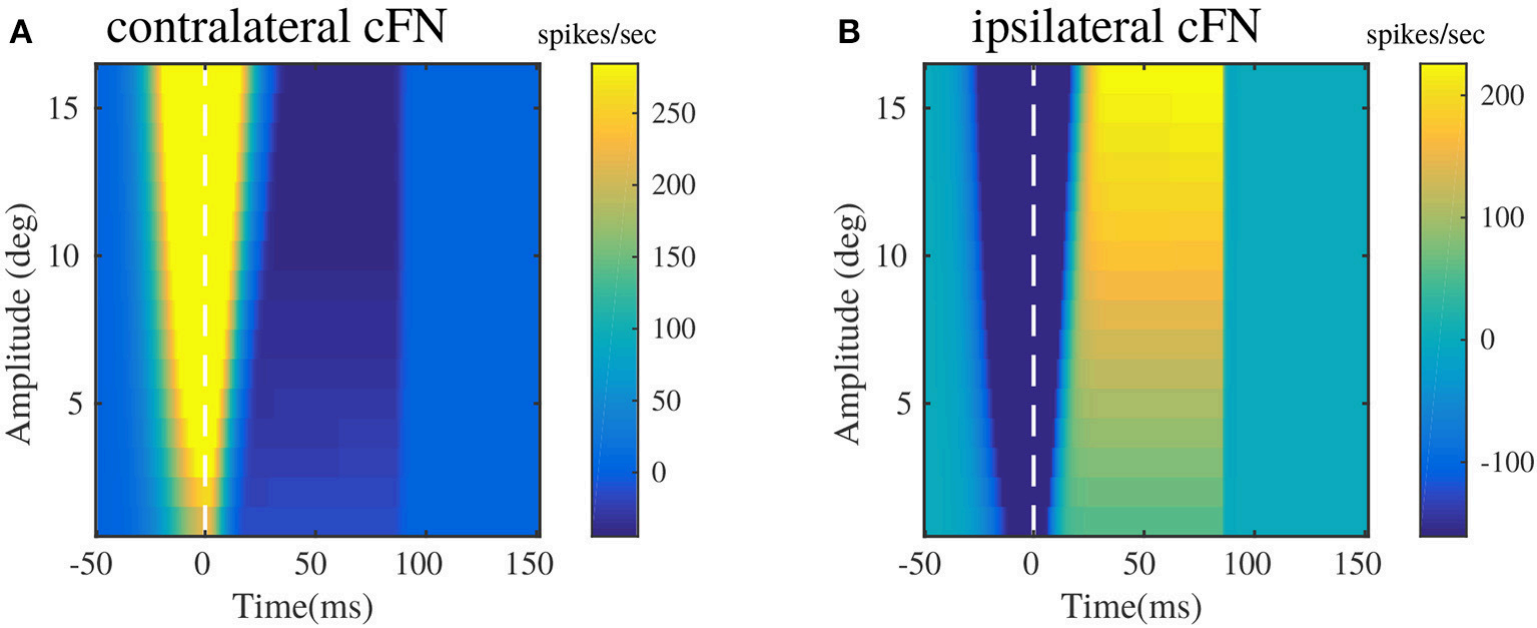
Bilateral cFN activities simulated for different saccade amplitudes in the range of 2–20°, post-adaptation. **(A)** Contralateral cFN activity is comprised of early burst in the activity above the baseline, whose intensity increases with the increase in desired target displacement, followed by pause in the activity. **(B)** Ipsilateral cFN activity is comprised of early pause, whose intensity can be observed to increase with increasing target displacement, followed by burst in the activity above the baseline.

Figure 5 illustrates the pattern of responses in the cFNs, post adaptation in the OMV. These patterns of activity provide feedback corrections to the burst generating units in the brainstem for different saccade amplitudes. The activities are represented as heat maps, to have a clear picture of the generated signals, for randomly chosen target locations between 5 and 20°. The baseline activities of cFN has been set to be at zero ordinate level, hence the degree of change in the color of the heat map directly represents the change in the activity with respect to the baseline. These results can be related to the pause-before-burst and burst-before-pause cFN unit activities observed in experimental recordings (Fuchs et al., 1993).

Bilateral cFN activity has been simulated to be the summation of activities from the PC populations, and the excitatory MF projections from the burst generating units. The resultant cFN activity is depicted in Figures 5A,B. Figure 5A illustrates that the contralateral cFN activity is characterized by a strong burst activity during the initialization of the saccadic movement, that forms a part of drive signal to the eye, as indicated in previous works (Fuchs et al., 1993; Gad and Anastasio, 2010). This burst is followed by a pause and subsequent return to the baseline activity (0 in this case), till the eye hold is triggered. Ipsilateral cFN signal activity is characterized by an early pause in the activity followed by a late burst above the baseline and subsequent return to the baseline (depicted in Figure 5B). In combination with the pause in contralateral cFN activity, this late ipsilateral cFN activity decelerates the eye from peak speed to a halt. It is worthy to note the high activity in the cFN units around the onset of eye movement (represented as dotted line at *t* = 0), whose intensity increases with the increase in saccade amplitude.

The experimental recordings on individual cFNs (Ohtsuka and Noda, 1991; Fuchs et al., 1993) indicate a modulation of burst duration in cFNs, while their peak burst remains constant. The current modeling results in Figure 5 depict an increasing burst duration with increasing saccade amplitudes, while the peak burst also increases slightly with the increase in saccade amplitude. In this regard, it is worthy to note that the current model describes the net contralateral and ipsilateral activity of cFN that project onto the brainstem MLBN system. The cFN activities depicted in Figures 5A,B are net population rate activities rather than individual cFN activities. Sufficient experimental observations are yet to be performed on the cummulative population activities of the cFN, that project onto each individual group of the distributed MLBNs with maximum burst discharge in a preferred movement direction.

**Figure 6:**
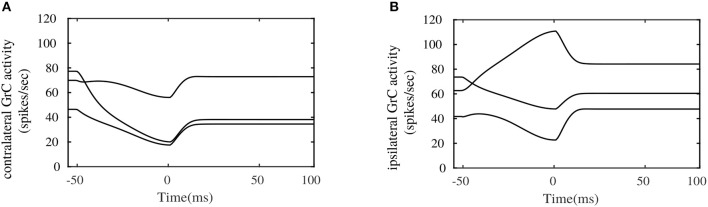
Simulated bilateral granule cell activities during the saccadic eye movement. **(A)** Activity in randomly picked granule cell units from the contralateral side. **(B)** Activity in randomly picked granule cell units from the ipsilateral side.

Figure 6 illustrates the pattern of activities in randomly picked granular layer units bilaterally. These plots show different responses in the amount of burst and pause generated within granular layer units on each side, during the eye movement. The combined activities of these granular layer units on each side provide temporal basis for the shaping of PC layer activities, and consequently for shaping the cFN activities on the respective sides. One important observation that could be made between the contralateral and ipsilateral granular units is the similarity in the temporal activity patterns between both sides, as depicted in Figures 6A,B. These activity patterns are characterized by relatively long temporal activity before the movement initiation at *t* = 0*ms* and a relatively short duration of increase or decrease in the activities during the movement. Performing visual error based learning in the PF-PC connection strengths, by considering the bilateral MFs to project onto a single side of granular layer (either ipsilateral or contralateral sides), yielded similar PC and cFN layer activity patterns as that of the bilateral granular layer configuration. The bilateral OMV outputs could, in principle, emerge from similar dynamics of the granular unit activities, by means of different PF-PC and MF_*burst*_-cFN synaptic strengths. This indicates that direction specific dynamics is not necessary in the granular layer. As the granular layer is composed of multiple cell types with specific roles and as few recordings are available, it is difficult to verify this argument.

### 3.4. OMV Compensations for Variabilities

Having simulated the model to generate sensory corrections, we chose to examine if the same model can explain the variabilities in eye trajectories observed during primate saccade adaptation experiments. In Xu-Wilson et al. (2009), the authors show that the participants with intact OMV are able to correct their movements against the variabilities in motor commands due to drop in motivational levels. The same was not valid for cerebellar patients, where the same structured variability resulted in saccade dysmetria.

We can simulate the reduction in saccade speeds in our model setup, by reducing the burst amplitude of the burst generating units, *A*, mentioned in internal feedback loop (see Equation 1). We perform simulation for the cases of an intact OMV that actively takes in the MF inputs and computes the compensations for the perceived reduction in motivation level (injected at the level of burst generating units), and a clamped OMV output which does not vary in relation to the variabilities in the burst generating units.

**Figure 7:**
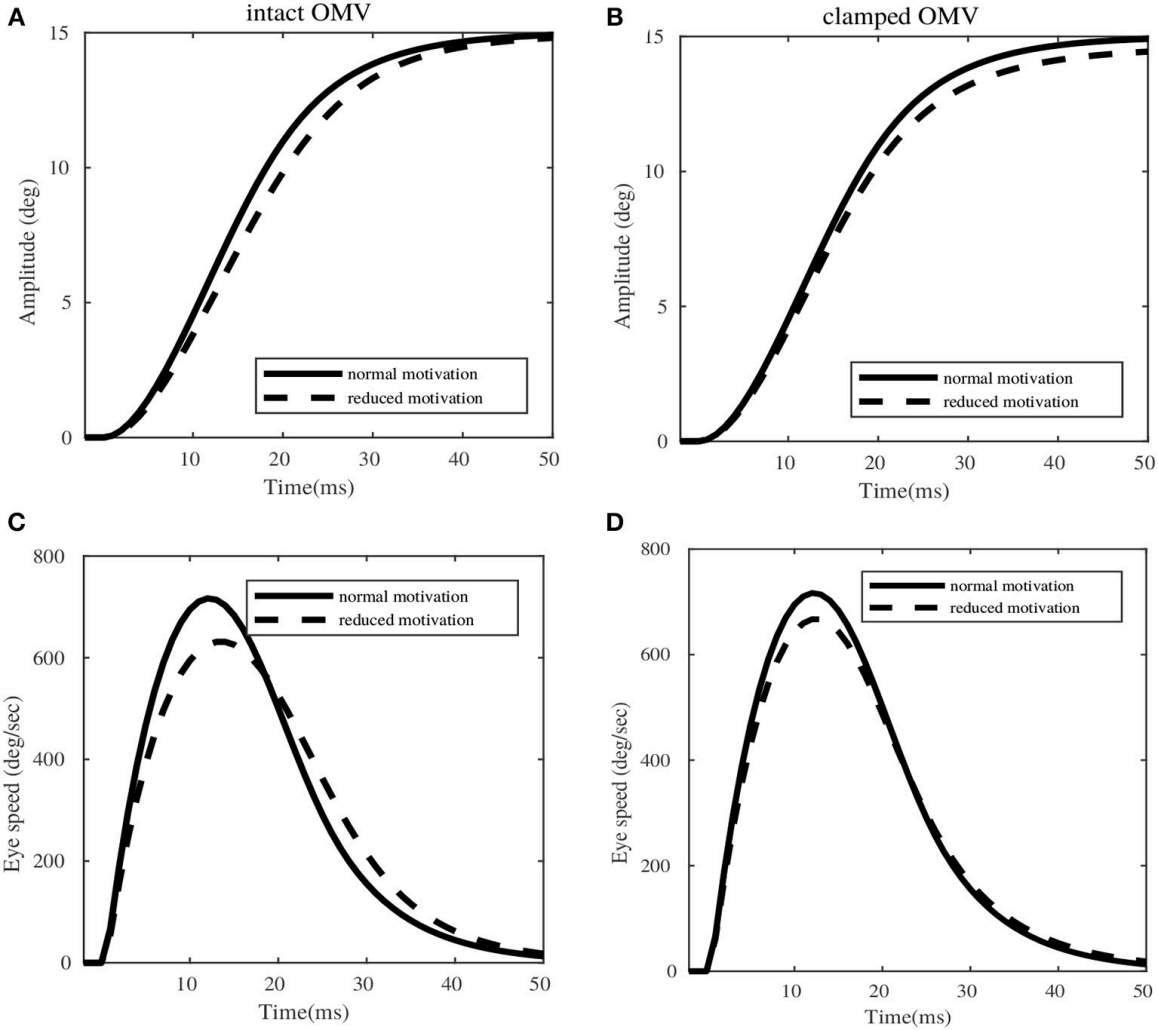
Simulations of eye movement profile for 15° target, in presence of variabilities in the extra-cerebellar pathway. **(A,C)** Represent the amplitude and speed variations with respect to normal and reduced motivational states for intact OMV. **(B,D)** Represent the amplitude and speed profiles with respect to normal and reduced motivational states for clamped OMV output.

Figure 7 illustrates the saccadic trajectories for a sample 15° desired target amplitude, with respect to intact OMV and clamped OMV output conditions. In the case of intact OMV (left column of Figure 7), the amplitude of the movement remains the same in both normal and reduced motivational states (induced by means of saccade burst reduction by 16%). However, a dysmetry of 2° is observed in the clamped OMV case (right column of Figure 7). While the peak velocity dropped in both the intact OMV and clamped OMV cases, only the intact OMV case showed a later correction for the reduced peak velocity. The intact OMV case showed a peak speed reduction of 11%, and the clamped OMV condition showed peak speed reduction of 14%, for the given target amplitude of 15°. However, this reduction in the speed was compensated by the corrective bulge observed in the speed profile, after 19*ms* (indicated by the dotted line), in the intact OMV trial. The same pronounced compensation at a later time cannot be seen in the clamped OMV output case, resulting in saccade dysmetria. It is important to note that, for these variability trials no further learning is required in the OMV. The previously learnt synaptic strength configuration is sufficient to provide compensation for variabilities. To do this, the OMV requires only information from the *MF*_*burst*_ projections.

## 4. Discussion

The principal outcome of this work, resulting from the proposed saccade model, is the definite encoding of saccade kinematic information in the combined burst-pause PC population response converging onto a common cFN. The total inhibitory activity from the PC layer on a given cFN, displayed a definite relationship with the speeds and amplitudes of the saccadic movements. The peak population response in the PCs increased linearly with the peak eye speeds, which corroborates the experimental evidences from Herzfeld et al. (2015). While emphasizing on the possible computations behind the observed PC population response, we have been able to reproduce stereotypical saccade amplitudes and speeds observed in monkeys (Bahill et al., 1975). However, we have observed a slight deviation from experimental observations (Herzfeld et al., 2015), in the relationship of the peak PC population response with the saccade amplitudes. While the experimental observations indicate a linear relationship of the peak PC population response with various desired saccade amplitudes, our model indicates a curved relationship. Combining the observation that saccade amplitudes and velocities are related in a similar curved manner, and the observed linear relationship between peak PC population response and saccade speeds, the non-linear curve between peak PC population response and saccade amplitudes should be a straightforward deduction. However, the experimental observations were presented in a restricted range of saccade amplitudes, from 10 to 15°, thereby could not have informed the possibility of non-linearities in a broader range of target amplitudes. Through our simulations, we indicate that the peak PC population responses are related to the saccade amplitudes, in a similar manner as that of the relationship between peak saccade speeds with amplitudes.

Further, our simulation results indicate the possible mechanism behind the OMV, in compensating for the inter-trial variability of saccade trajectories associated with the motivational states of the primates. In the experimental studies conducted on humans (Xu-Wilson et al., 2009), the authors demonstrated a drop in the eye speeds along increasing number of trials, as the human subjects repeatedly executed saccades to a given target location. Appropriate corrections in motor commands were supposed to be provided by the OMV at the end of the eye movement, to counter the initialization at lower speeds. However, the neural correlates of this phenomenon were not completely clear. Variabilities can be injected into multiple sites of saccade production model, consequently having specific effects on the output trajectories (Eggert et al., 2016). For example, motivational state of the primates, corresponding to rewarded or non-rewarded target locations, is observed to induce variabilities in saccade velocities (Kawagoe et al., 1998). This is possible due to the variable importance assigned to specific target locations, by means of the distributed neural connections in basal ganglia (Hikosaka et al., 2000; Kawagoe et al., 2004), projecting onto the superior colliculus (SC). To examine the specific case of speed reduction presented in Xu-Wilson et al. (2009), we induced an amplitude reduction in the burst characteristics of the internal feedback loop. By means of our simulations we observed that, the OMV does not need to undergo further **w**_*pf*−*pc*_ connection strength adaptation inorder to associate different motivational states of the primate with different PC population responses. The OMV acquires forward estimation of sensory consequences as a function of target displacement, current state of the burst generator and eye position. Variabilities in the burst responses of the internal feedback loop are reflected in the information carried by mossy fibers, arising from the burst generating units. This mechanism automatically provides an input state separation, which the presented OMV organization is able exploit to generate appropriate trajectory corrections. The same compensatory mechanism was not observed in the clamped OMV simulation.

Granular layer plays an important role in expanding the given mossy fiber input states into complex and sparse information, to provide appropriate activity at the PC layer. The dominant neuronal units observed in the granule layer, are the granule cells (GrCs) and golgi cells (GCs). Our current model is agnostic to the distinct GrC and GC populations, and considers a population of random-sparse-recurrent inhibitory connections between uniform granular layer units. This kind of granular layer computation, is inspired from the proposed computational studies in Yamazaki and Nagao (2012), Rössert et al. (2015), and Bratby et al. (2016). Further, we have implemented granular layer in both contralteral and ipsilateral directions, with different interconnections as represented in Figure 2. The purpose of considering bilateral granular layer projections was to examine if distinct patterns of granule unit activities emerge for generating the necessary bilateral PC layer activities. Experimental observations in Prsa et al. (2009), on the GC activities during saccadic eye movements indicate that several directions of movement are represented equally in the population responses of GCs, without any directional selectivity. This directional independence in the GCs is in contrast with the strong directional preferences observed in the MF discharges related to saccades (Kase et al., 1980; Ohtsuka and Noda, 1992). By our simulations, we suggest that similar patterns of granular activities can indeed produce both contralateral and ipsilateral PC layer activities (CS-on and CS-off PC layers, respectively), by means of different PF-PC synaptic strengths. Hence, direction specific dynamics seems to be not necessary in the granular layer, in order to induce appropriate activities in the Purkinje layer for precise eye movement control.

The plastic PF-PC weights, have been modulated by a visual error teaching signal. Unlike motor tasks such as vestibulo-ocular reflex and simple eye blink conditioning, the desired output of the cerebellum from the deep cerebellar nuclei does not need to be directly available from the target information, but could be a result of an unreferenced motor control problem (Harris, 1998). However, through our simulations, we display the applicability of sensory error information, which is not immediate but distal (Dean et al., 2002), to enable appropriate adaptation of the plastic synaptic sites in the OMV. The nature of the teaching signal determines whether the output from the cerebellum should compensate for sensory error signals (Tseng et al., 2007), or motor correction signals (Kawato and Gomi, 1992). In the former case, the cerebellum should provide feedback compensation in the sensory space (function of cerebellum as a *forward model*). In the latter case, the cerebellum could function as an *inverse model*, that provides feedforward compensation in the motor space. Though the forward model function of cerebellum has been hypothesized to have computational advantages in a number of studies (Dean et al., 2002, 2010), each specific motor control task still requires enough computational and experimental evidence, to validate the relevance of the two view points (Iwamoto and Kaku, 2010). In case of saccade adaptation, experimental evidence from stimulation studies indicates that the adaptation could take place through visual error information, even in the absence of corrective motor actions (Wallman and Fuchs, 1998). We further investigated this view by our computational model. However it should be noted that, though saccade adaptation is likely to be guided by visual cells in the SC (Soetedjo et al., 2009), there is a possibility that deeper layers of the SC have additional encoding of motor error, hence aiding an inverse model adaptation. In compliance with the experimental observations on rhesus monkeys in Herzfeld et al. (2015), our simulations foster the hypothesis of forward sensory prediction in the OMV compensations. A further elucidation regarding this prospect, for different stages of primate development, needs more experimental evidence.

The fundamental unit of cerebellar computation can be considered to consist of a repetitive pattern of connectivity, called microzone (Dean et al., 2010; Jörntell, 2017). The computational properties of this microzone arrangement have been extensively studied, pertaining to the flocculus region of the cerebellum, through VOR (Ito, 1998; Clopath et al., 2014) and eye-blink conditioning responses (Herreros and Verschure, 2013). It is important to understand the nature of cerebellar microzones in complex motor control contexts like saccades. This could enable us to understand the way these microzones are organized in the larger scheme of sensory-motor control, with various levels of adaptation and decision making. However, a considerable understanding of the OMV, which is related to saccade motor control, has been limited until now, due to the presence of multiple sites and time scales of saccade adaptation. The saccade adaptation can be studied in the context of long-term adaptation (Robinson et al., 2006) that takes place over duration of several days, and short-term adaptation (Hopp and Fuchs, 2004) that takes place over durations of several hours. According to recent experimental observations, the short adaptation could be further divided into two more time scales, at the direct and indirect pathways of saccade production (Chen-Harris et al., 2008). The adaptation process that we addressed in this paper corresponds to the plasticity in the indirect saccade pathway, corresponding to the OMV and cFN components. Through our simulation results, we observe that a definite encoding in the PC layer activity of the OMV, could enable precise online endpoint control in the presence of variabilities in the cortical control loop. However, experimental studies indicate that lesions in the OMV do not necessarily warrant long-term inaccuracies (Barash et al., 1999), thus indicating other possible sites of adaptation. Another model proposed in Saeb et al. (2011) could be explored for a plausible candidate for adaptation in the direct pathway, corresponding to the SC and MLBN connections. In any case, it would be an interesting direction to have a comprehensive model, that could explain multiple timescales of adaptation. This extension could provide additional insights into the organization of the OMV, and the kind cerebellar connectivity that could be responsible for multiple timescales of adaptation.

Though the implementation of timing dependent plasticity at the plastic synaptic connections (Schweighofer et al., 1996; Gad and Anastasio, 2010) could throw light on the rate of learning from visual errors, we simplify the adaptation procedure with numerical gradient estimation, to focus on the control objectives of saccade adaptation. However, the learning criterion used to minimize the visual error, is indeed conducive for the derivation of incremental gradient based learning rules presented in Dean et al. (2002) and Saeb et al. (2011). The recurrent, nonlinear inhibitory connections in the granular layer do not impede the application of such incremental linear approximation based learning rules, as evident from Carrillo et al. (2008) and Casellato et al. (2014). This is because of the liquid state property of the cerebellar granular layer (Yamazaki and Tanaka, 2007a), which enables learning of complex motor actions, only by adjusting the linear readout synaptic strengths (Sussillo and Abbott, 2009; Rössert et al., 2015; Bratby et al., 2016). One property that we could not comment upon, by the very nature of a control block simplification of the OMV, is the full-scale directional specificity in the PC population activity (Soetedjo et al., 2008). The Purkinje cells of the OMV are organized to receive MF inputs from all the sites in the SC (Fujita, 2005). However, the adaptation in these PCs is driven by teaching signals received from climbing fiber (CF) afferents, with a definite directional specificity (Soetedjo et al., 2008). The PCs receiving similar CF information are further hypothesized to be projected onto specific cFNs (Herzfeld et al., 2015). Hence, the adaptation for a given saccade vector is specific to certain PC populations, and regulated by the CF afferents of that particular directional specificity (Iwamoto and Kaku, 2010). This direction specific adaptation in the OMV was referred to as parametric adaptation in Hopp and Fuchs (2004). Due to the compact representation of the entire OMV as simplified control blocks, we could examine the ipsilateral and contralateral sides of movement but not the whole topographic space. Hence it was not possible to analyze the parametric adaptation. However, the gap between our current model and a study of directional specificity in PC layer activity can be overcome, by implementing an increased number of directionally tuned teaching signals. We found out through a number of trials that expanding the error reduction methodology used in this paper for parametric adaptation is computationally expensive, due to the increase in number of plastic synaptic connections to be optimized, and requires an implementation of incremental plasticity rules.

## Data Availability Statement

The raw data supporting the conclusions of this manuscript will be made available by the authors, without undue reservation, to any qualified researcher.

## Author Contributions

HK, TG, EF, and CL conceived and designed the experiments and wrote the paper. HK performed the experiments and analyzed the data.

### Conflict of Interest Statement

The authors declare that the research was conducted in the absence of any commercial or financial relationships that could be construed as a potential conflict of interest.
